# Supplementary material for: Transcriptome Analysis of Goat Mammary Gland Tissue Reveals the Adaptive Strategies and Molecular Mechanisms of Lactation and Involution
Source: Int J Mol Sci. 2022 Nov 20;23(22):14424. doi: 10.3390/ijms232214424 (PMC9693614; doi:10.3390/ijms232214424)
Supplement: Supplementary file 1 [file ijms-23-14424-s001.zip › Supplementary Figures.pdf]

## Supplementary Materials Title

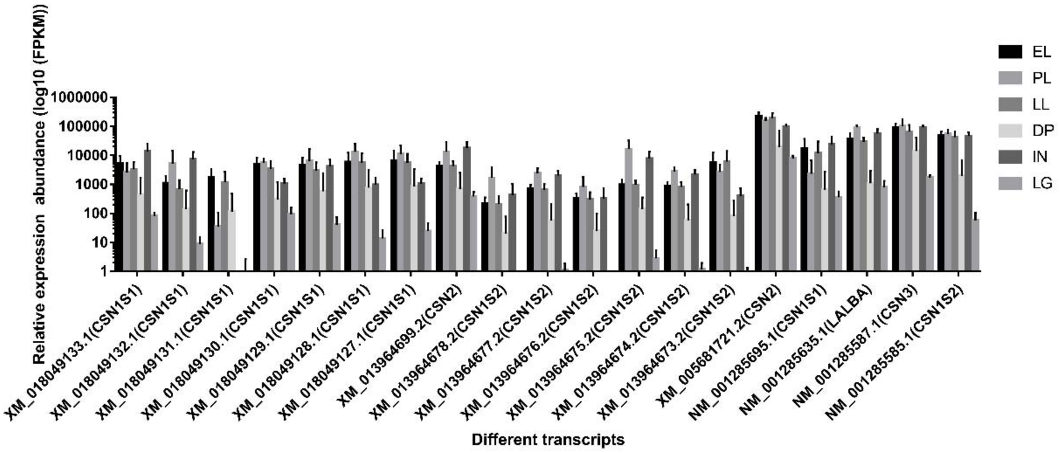


**Figure S1.** Expression levels of genes encoding milk proteins in mammary gland tissues at six developmental stages. EL: early lactation. PL: peak lactation. LL: late lactation. DP: dry period. IN: involution stage. LG: late gestation.

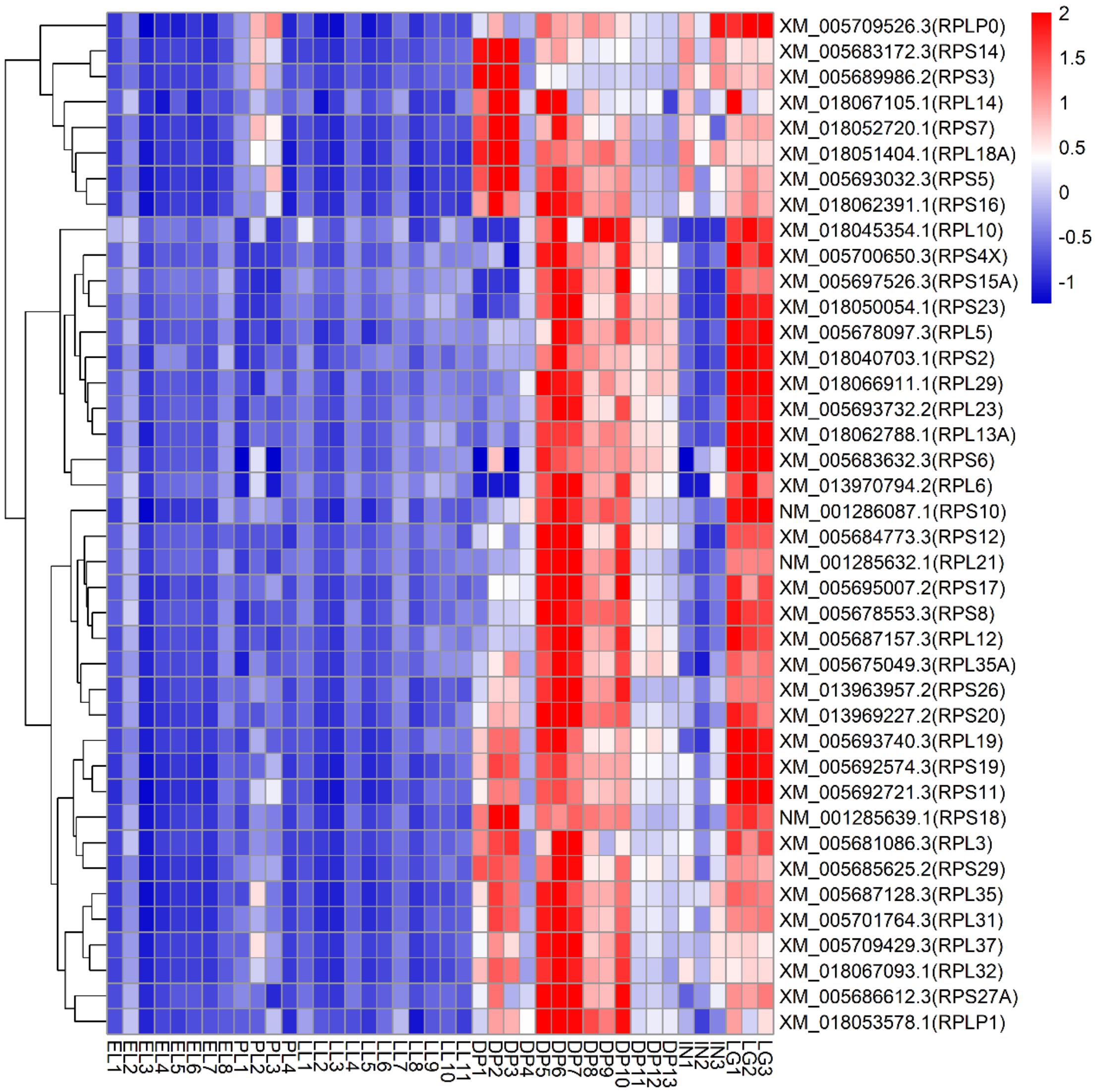


**Figure S2.** Expression levels of genes encoding ribosomes in mammary gland tissues at six developmental stages. EL: early lactation. PL: peak lactation. LL: late lactation. DP: dry period. IN: involution stage. LG: late gestation.

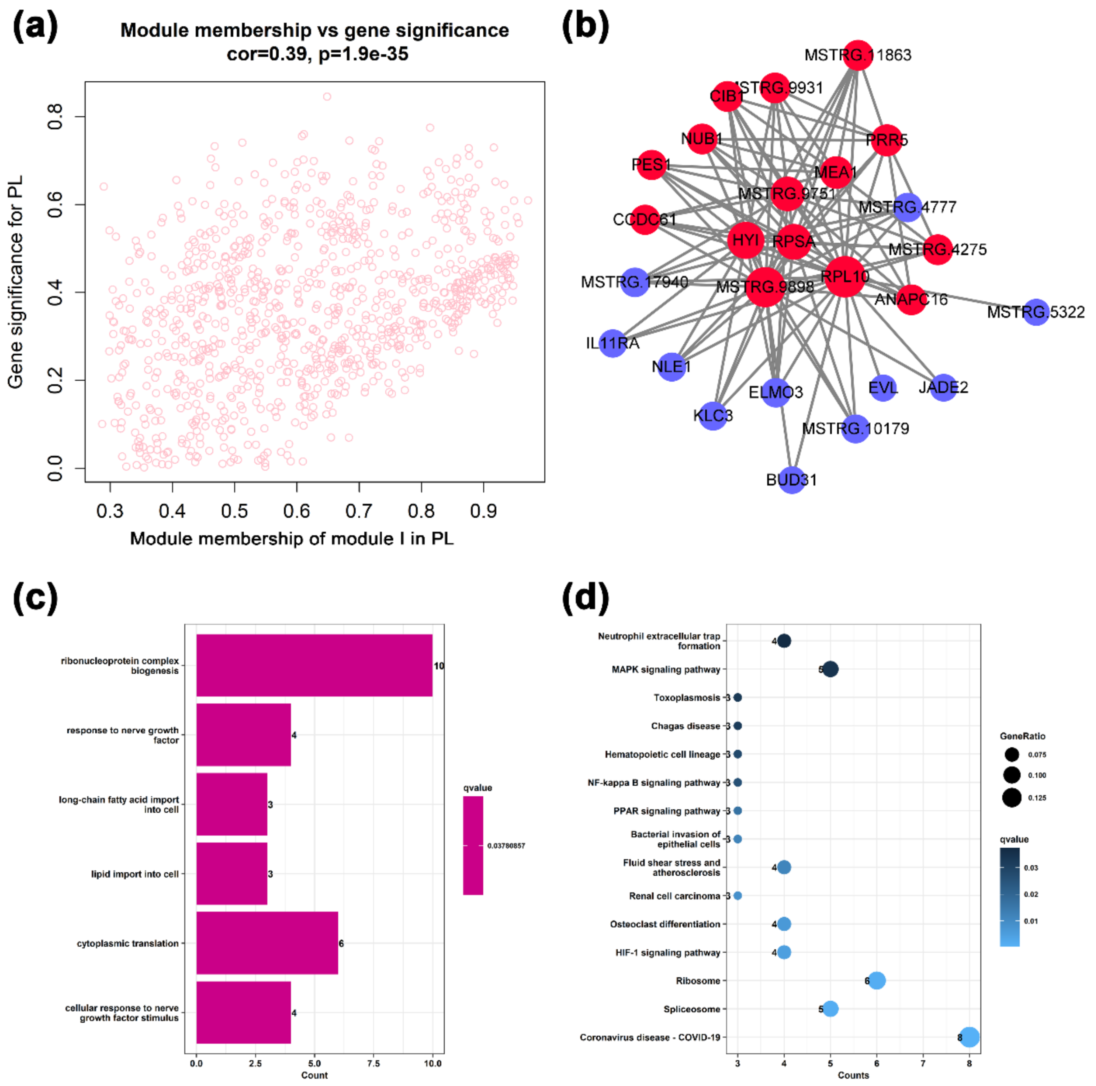


**Figure S3.** Pink module gene functional analysis. (**a**) A scatterplot of Gene Significance (GS) for the peak lactation (PL) stage vs. Module Membership (MM) in the pink module. There is a significant correlation (cor = 0.39, $p < 0.05$) between GS and MM in this module. (**b**) Visualization of connections of genes in the pink module. According to the connection weight value, only the first 100 functional relationships are shown in the figure. (**c**) Gene Ontology (GO) enrichment analysis of genes in the pink module associated with the PL. (**d**) Kyoto Encyclopedia of Genes and Genomes (KEGG) pathway enrichment analysis of genes in the pink module associated with the PL.

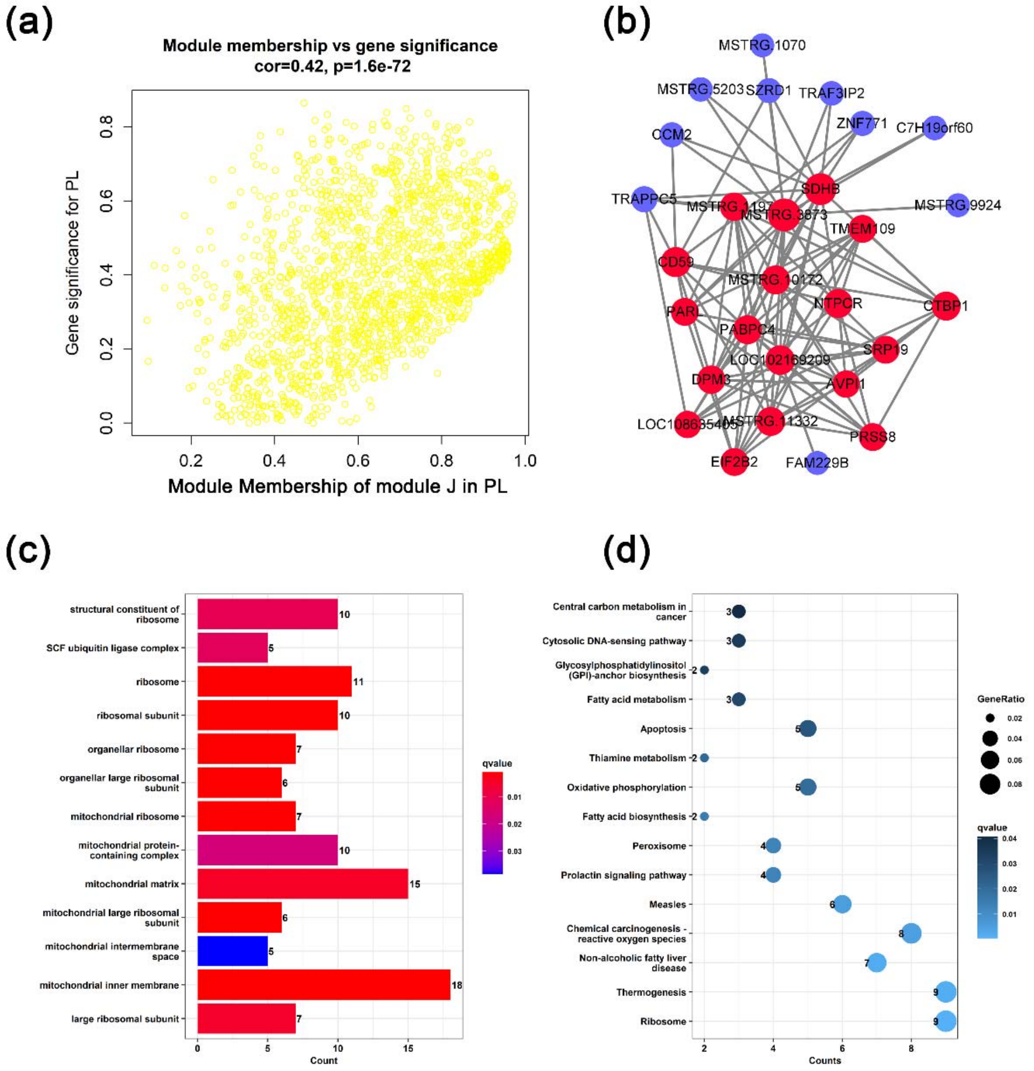


**Figure S4.** Yellow module gene functional analysis. (**a**) A scatterplot of Gene Significance (GS) for the peak lactation (PL) stage vs. Module Membership (MM) in the yellow module. There is a significant correlation (cor = 0.42, $p < 0.05$) between GS and MM in this module. (**b**) Visualization of connections of genes in the yellow module. According to the connection weight value, only the first 100 functional relationships are shown in the figure. (**c**) Gene Ontology (GO) enrichment analysis of genes in the yellow module associated with the PL. (d) Kyoto Encyclopedia of Genes and Genomes (KEGG) pathway enrichment analysis of genes in the yellow module associated with the PL.

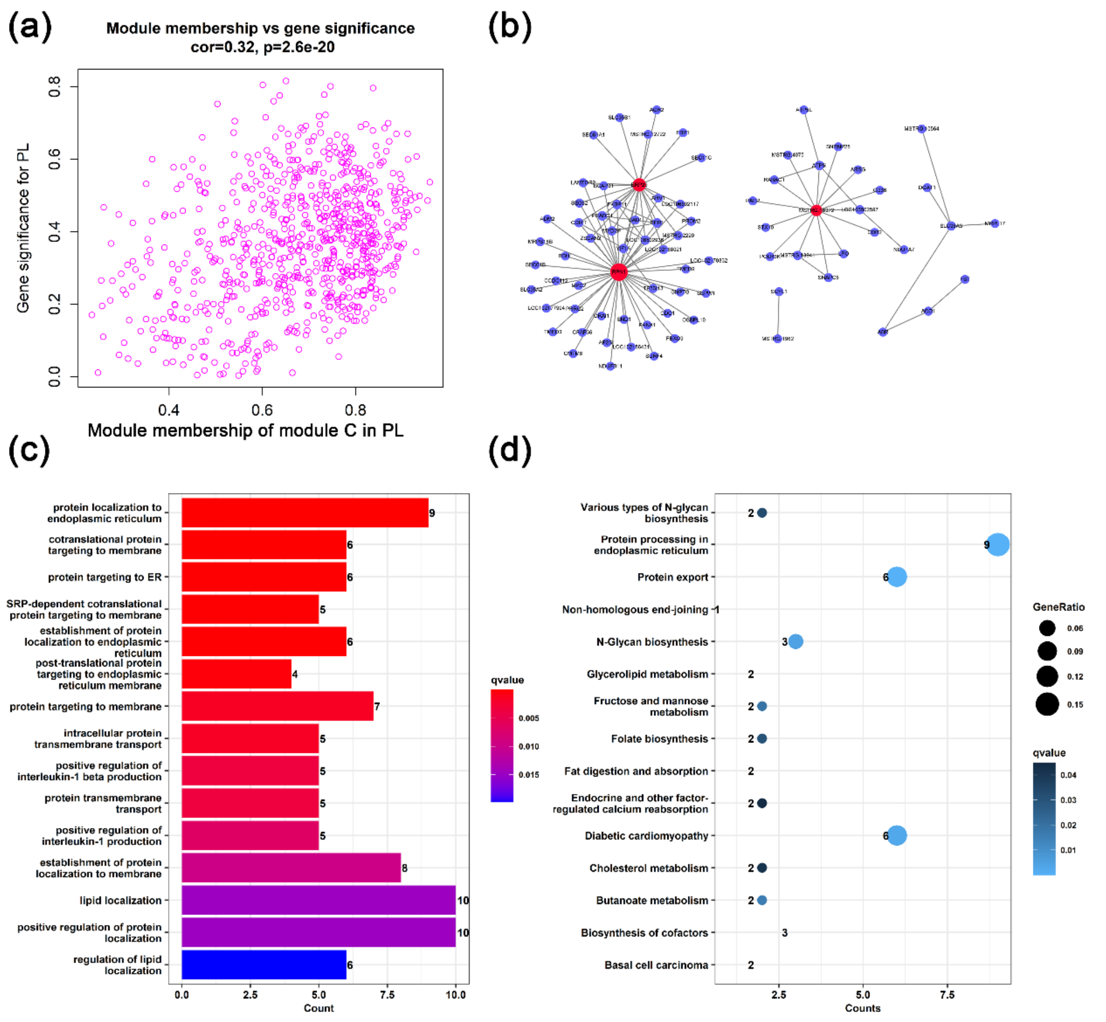


**Figure S5.** Magenta module gene functional analysis. (**a**) A scatterplot of Gene Significance (GS) for the peak lactation (PL) stage vs. Module Membership (MM) in the magenta module. There is a significant correlation (cor = 0.32, $p < 0.05$) between GS and MM in this module. (**b**) Visualization of connections of genes in the magenta module. According to the connection weight value, only the first 100 functional relationships are shown in the figure. (**c**) Gene Ontology (GO) enrichment analysis of genes in the magenta module associated with the PL. (d) Kyoto Encyclopedia of Genes and Genomes (KEGG) pathway enrichment analysis of genes in the magenta module associated with the PL.

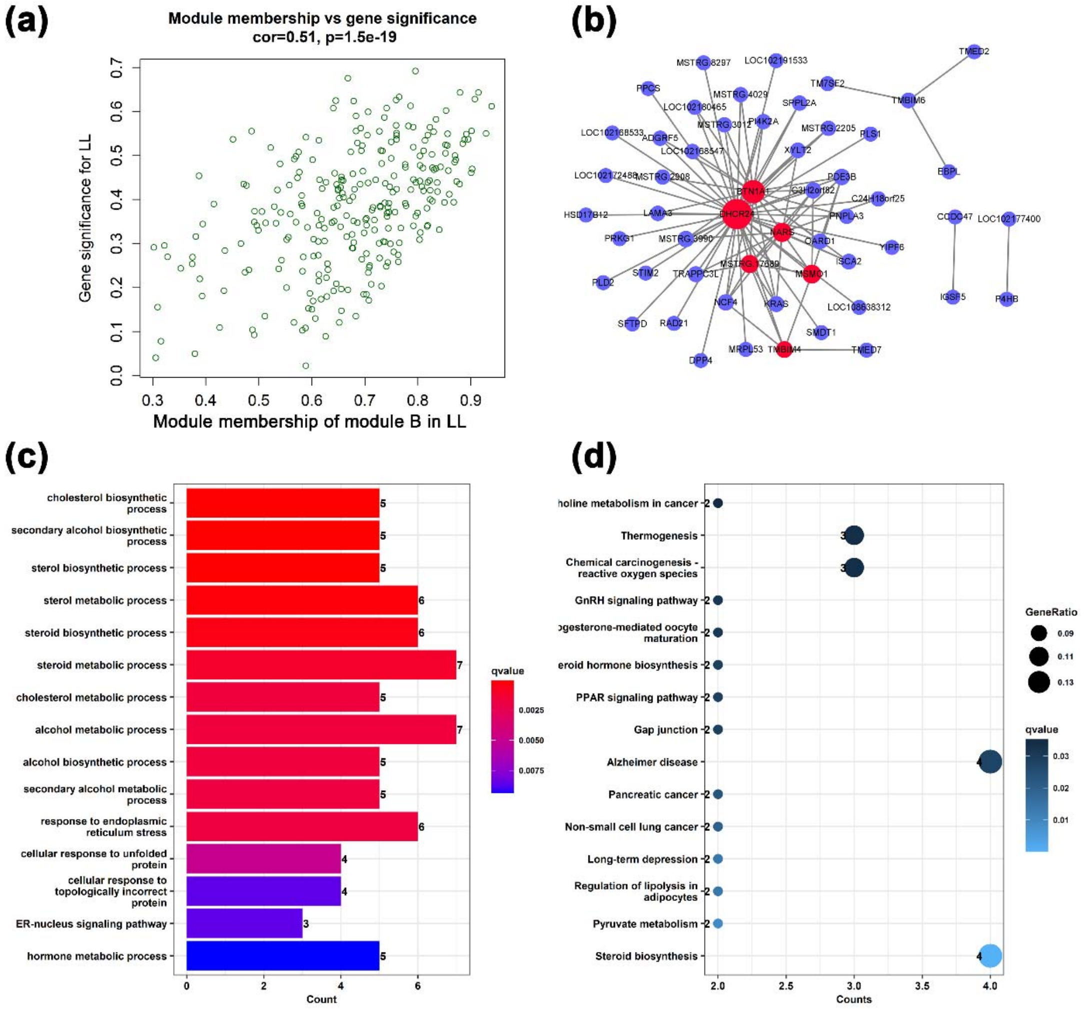


**Figure S6.** Darkgreen module gene functional analysis. (**a**) A scatterplot of Gene Significance (GS) for the late lactation (LL) stage vs. Module Membership (MM) in the darkgreen module. There is a significant correlation (cor = 0.51, $p < 0.05$) between GS and MM in this module. (**b**) Visualization of connections of genes in the darkgreen module. According to the connection weight value, only the first 100 functional relationships are shown in the figure. (**c**) Gene Ontology (GO) enrichment analysis of genes in the darkgreen module associated with the LL. (d) Kyoto Encyclopedia of Genes and Genomes (KEGG) pathway enrichment analysis of genes in the yellow module associated with the LL.

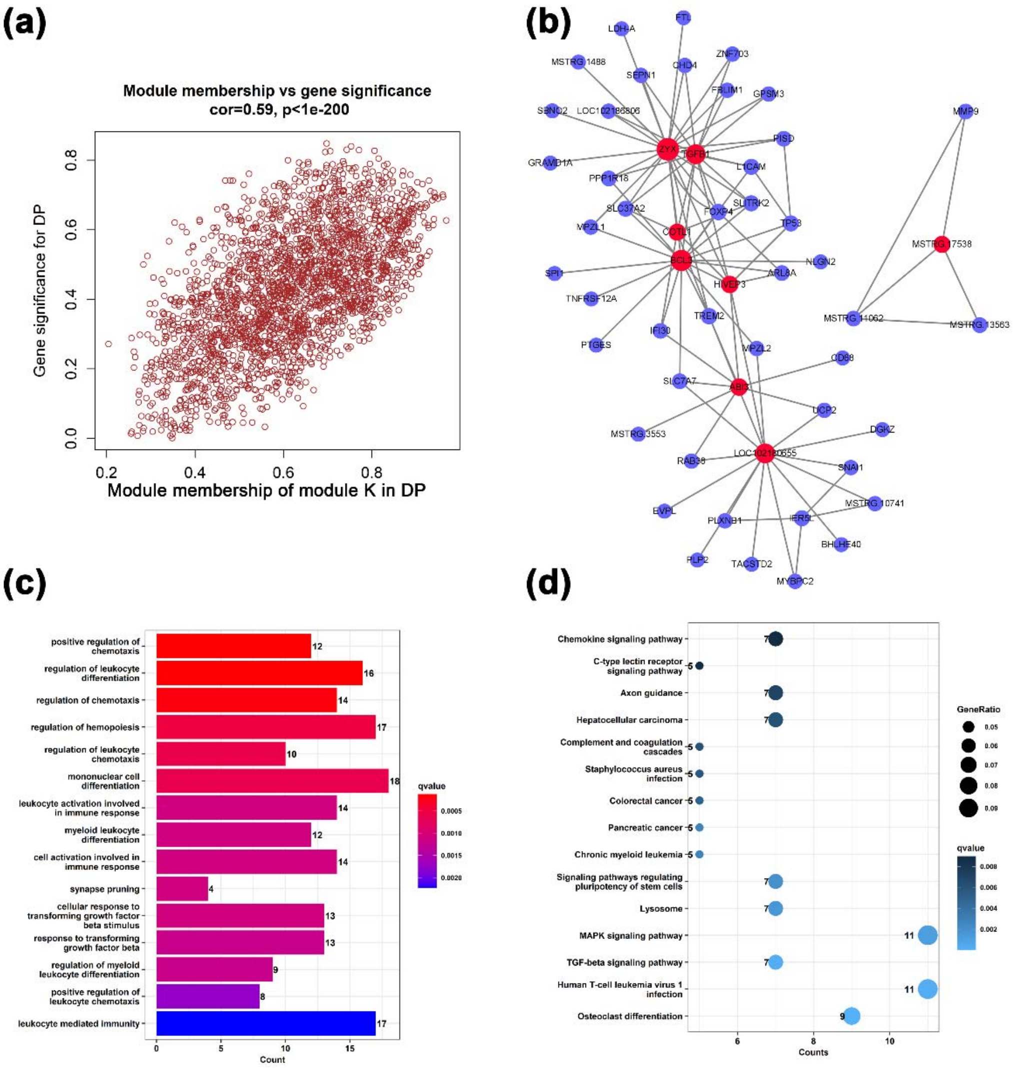


**Figure S7.** Brown module gene functional analysis. (**a**) A scatterplot of Gene Significance (GS) for the dry period (DP) stage vs. Module Membership (MM) in the brown module. There is a significant correlation (cor = 0.59, $p < 0.05$) between GS and MM in this module. (**b**) Visualization of connections of genes in the brown module. According to the connection weight value, only the first 100 functional relationships are shown in the figure. (**c**) Gene Ontology (GO) enrichment analysis of genes in the brown module associated with the DP. (d) Kyoto Encyclopedia of Genes and Genomes (KEGG) pathway enrichment analysis of genes in the brown module associated with the DP.

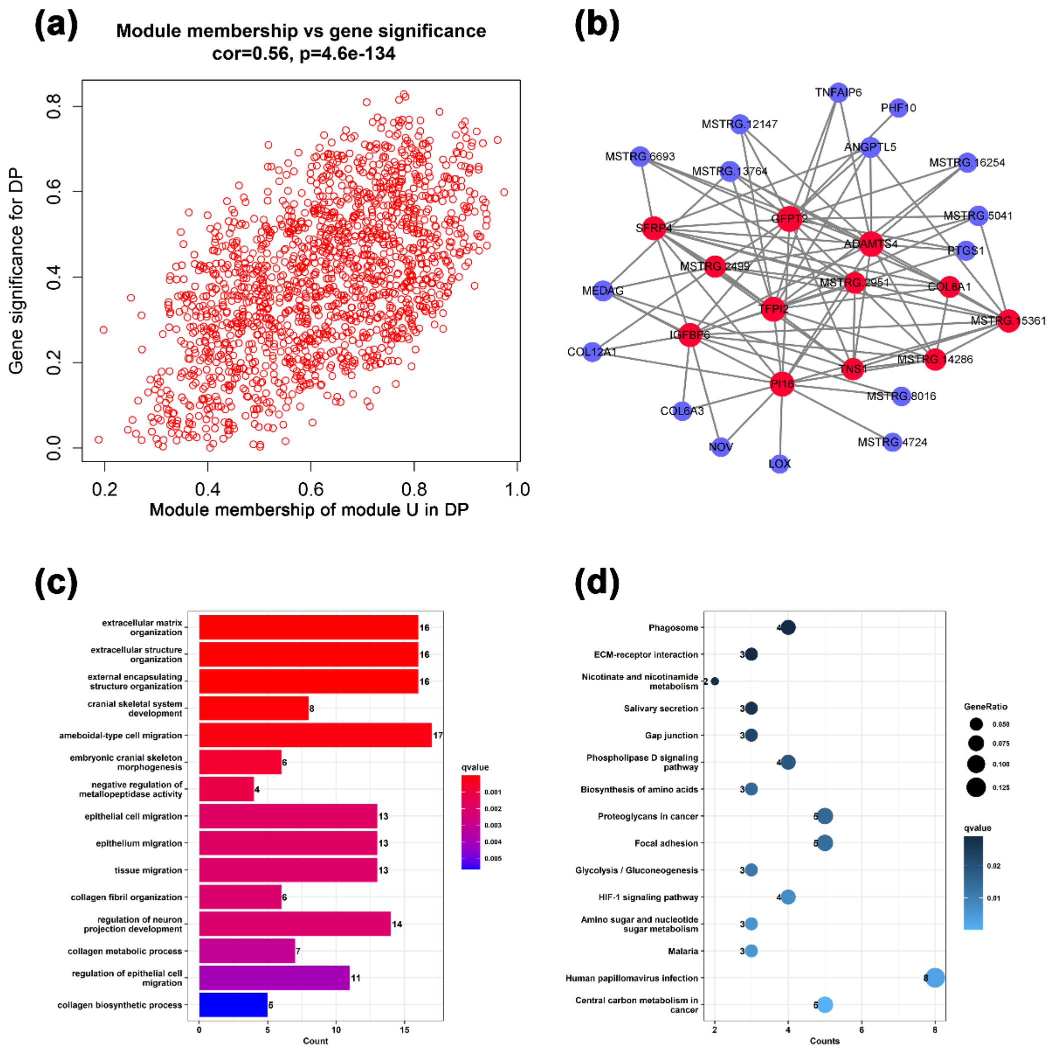


**Figure S8.** Red module gene functional analysis. (**a**) A scatterplot of Gene Significance (GS) for the dry period (DP) vs. Module Membership (MM) in the red module. There is a significant correlation (cor = 0.56, $p < 0.05$) between GS and MM in this module. (**b**) Visualization of connections of genes in the red module. According to the connection weight value, only the first 100 functional relationships are shown in the figure. (**c**) Gene Ontology (GO) enrichment analysis of genes in the red module associated with the DP. (**d**) Kyoto Encyclopedia of Genes and Genomes (KEGG) pathway enrichment analysis of genes in the red module associated with the DP.

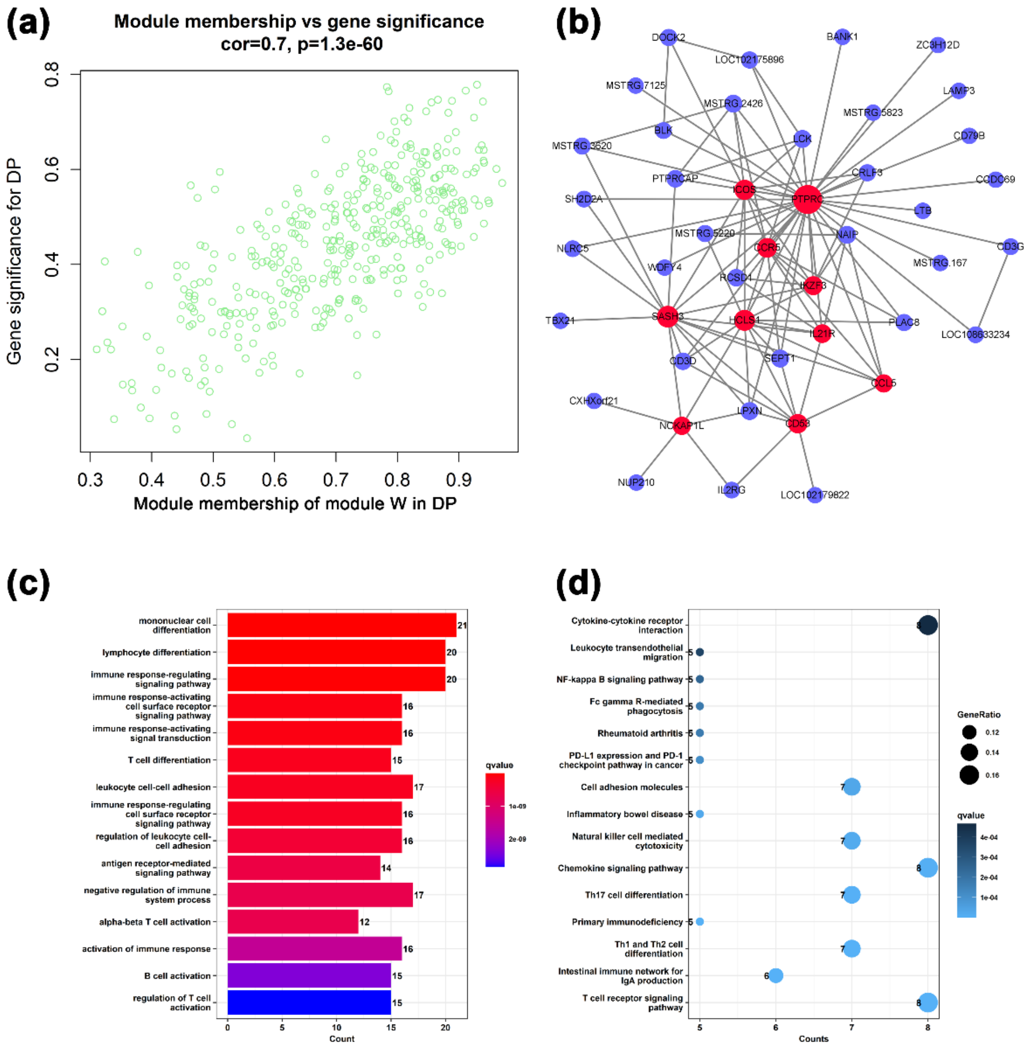


**Figure S9.** Lightgreen module gene functional analysis. (**a**) A scatterplot of Gene Significance (GS) for the dry period (DP) vs. Module Membership (MM) in the lightgreen module. There is a significant correlation (cor = 0.7, $p < 0.05$) between GS and MM in this module. (**b**) Visualization of connections of genes in the lightgreen module. According to the connection weight value, only the first 100 functional relationships are shown in the figure. (**c**) Gene Ontology (GO) enrichment analysis of genes in the lightgreen module associated with the DP. (**d**) Kyoto Encyclopedia of Genes and Genomes (KEGG) pathway enrichment analysis of genes in the lightgreen module associated with the DP.

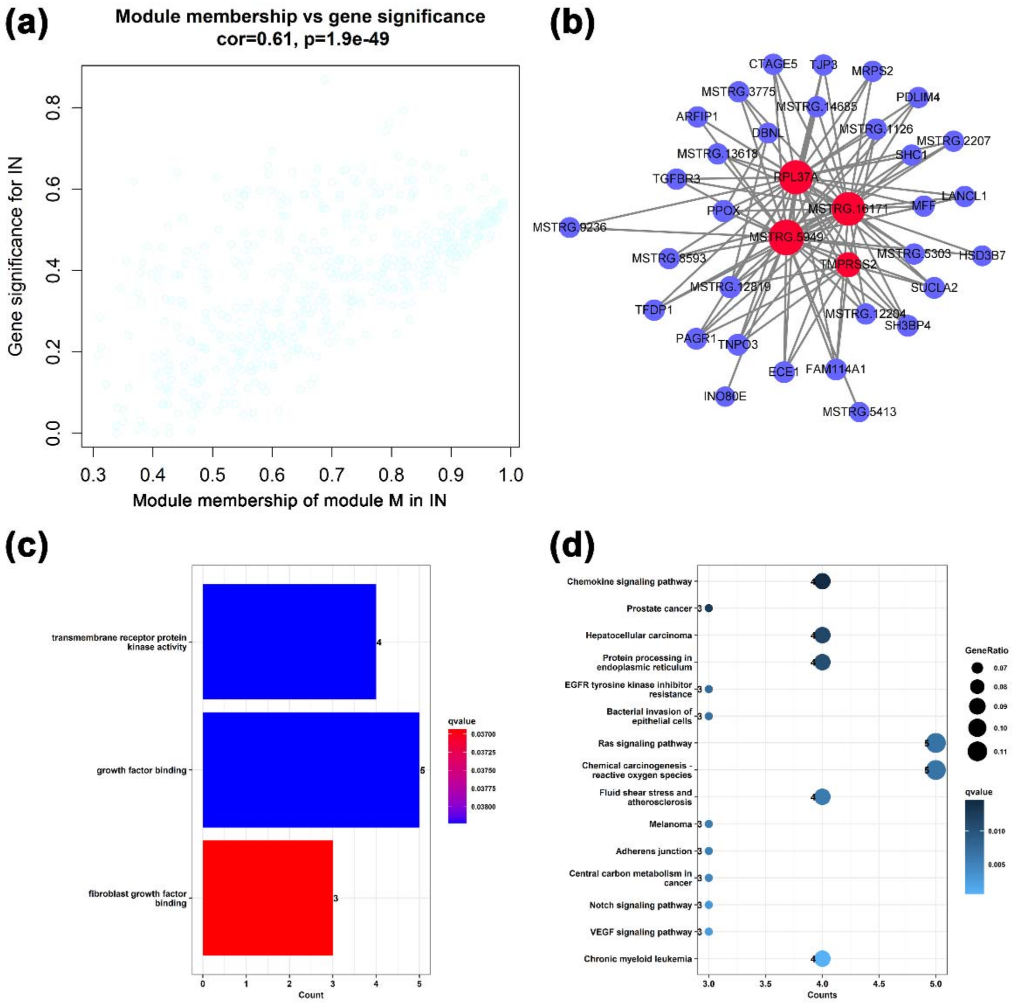


**Figure S10.** Lightcyan module gene functional analysis. (**a**) A scatterplot of Gene Significance (GS) for the involution (IN) stage vs. Module Membership (MM) in the lightcyan module. There is a significant correlation (cor = 0.61, $p < 0.05$) between GS and MM in this module. (**b**) Visualization of connections of genes in the lightcyan module. According to the connection weight value, only the first 100 functional relationships are shown in the figure. (**c**) Gene Ontology (GO) enrichment analysis of genes in the lightcyan module associated with the IN. (**d**) Kyoto Encyclopedia of Genes and Genomes (KEGG) pathway enrichment analysis of genes in the lightcyan module associated with the IN.

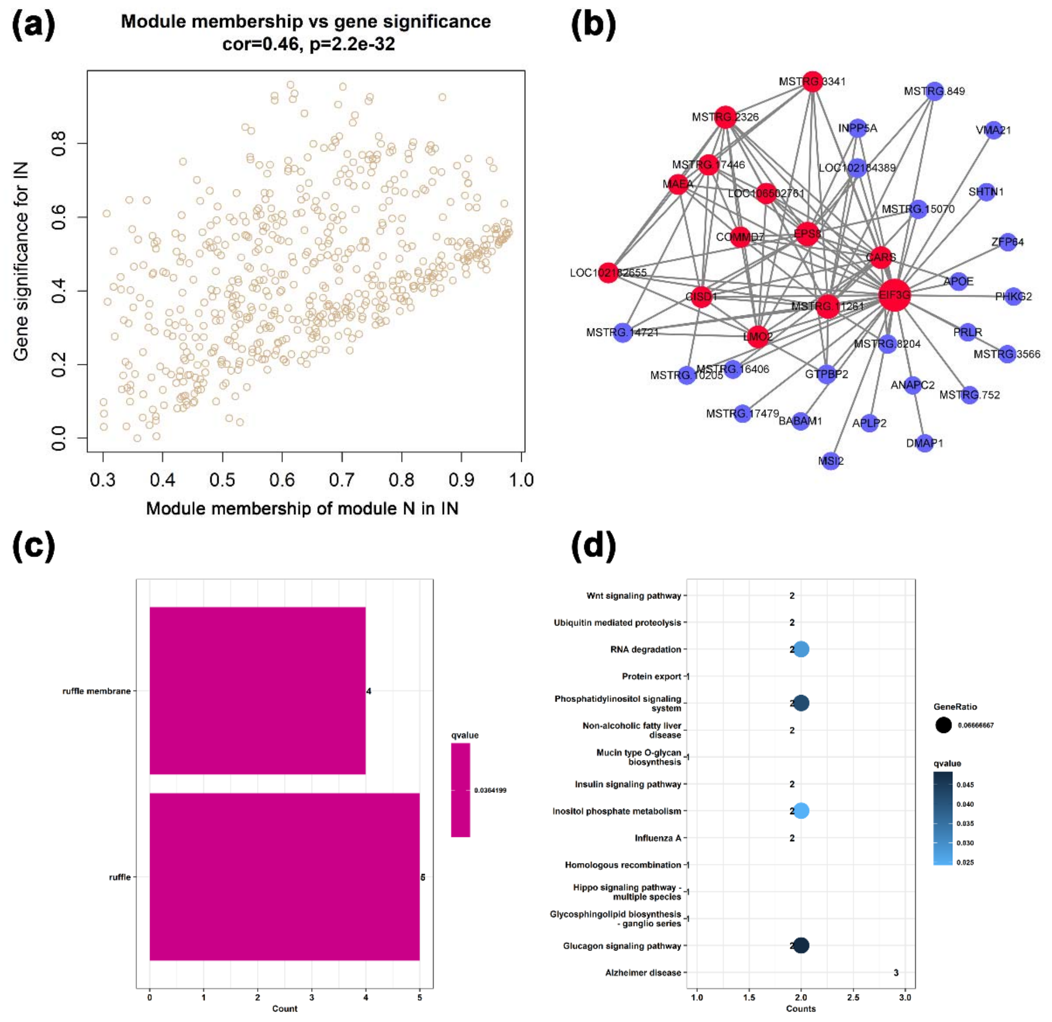


**Figure S11.** Tan module gene functional analysis. (**a**) A scatterplot of Gene Significance (GS) for the involution (IN) stage vs. Module Membership (MM) in the tan module. There is a significant correlation (cor = 0.46, $p < 0.05$) between GS and MM in this module. (**b**) Visualization of connections of genes in the tan module. According to the connection weight value, only the first 100 functional relationships are shown in the figure. (**c**) Gene Ontology (GO) enrichment analysis of genes in the tan module associated with the IN. (**d**) Kyoto Encyclopedia of Genes and Genomes (KEGG) pathway enrichment analysis of genes in the tan module associated with the IN.

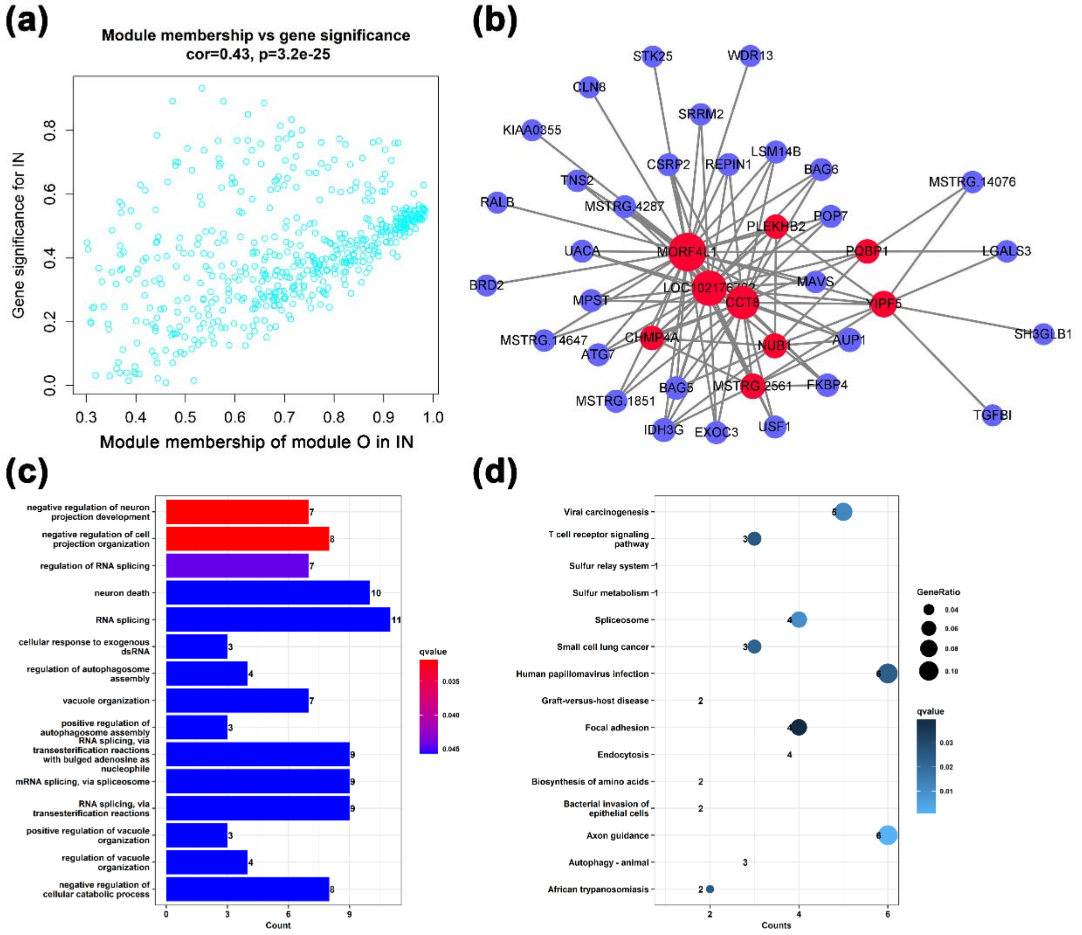


**Figure S12.** Cyan module gene functional analysis. (**a**) A scatterplot of Gene Significance (GS) for the involution (IN) stage vs. Module Membership (MM) in the cyan module. There is a significant correlation (cor = 0.43, $p < 0.05$) between GS and MM in this module. (**b**) Visualization of connections of genes in the cyan module. According to the connection weight value, only the first 100 functional relationships are shown in the figure. (**c**) Gene Ontology (GO) enrichment analysis of genes in the cyan module associated with the IN. (**d**) Kyoto Encyclopedia of Genes and Genomes (KEGG) pathway enrichment analysis of genes in the cyan module associated with the IN.

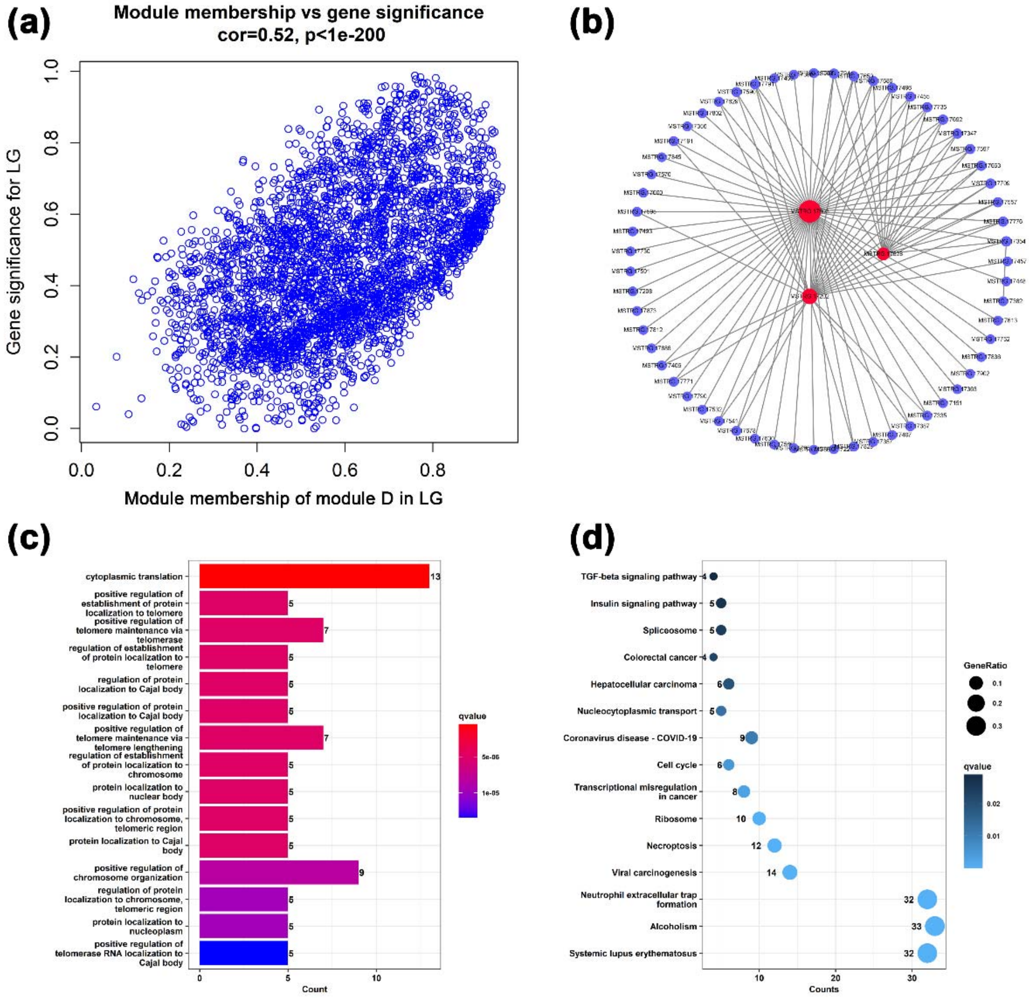


**Figure S13.** Blue module gene functional analysis. (**a**) A scatterplot of Gene Significance (GS) for the late gestation (LG) stage vs. Module Membership (MM) in the blue module. There is a significant correlation (cor = 0.52, $p < 0.05$) between GS and MM in this module. (**b**) Visualization of connections of genes in the blue module. According to the connection weight value, only the first 100 functional relationships are shown in the figure. (**c**) Gene Ontology (GO) enrichment analysis of genes in the blue module associated with the LG. (**d**) Kyoto Encyclopedia of Genes and Genomes (KEGG) pathway enrichment analysis of genes in the blue module associated with the LG.

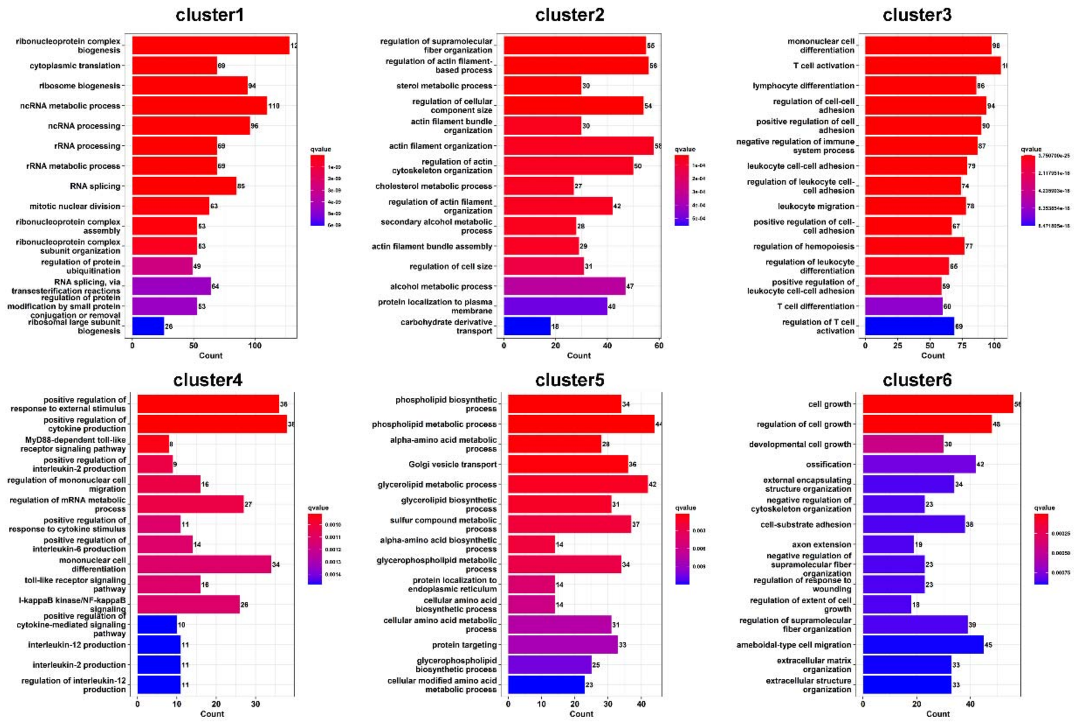


**Figure S14.** Gene ontology enrichment analysis of different cluster genes.

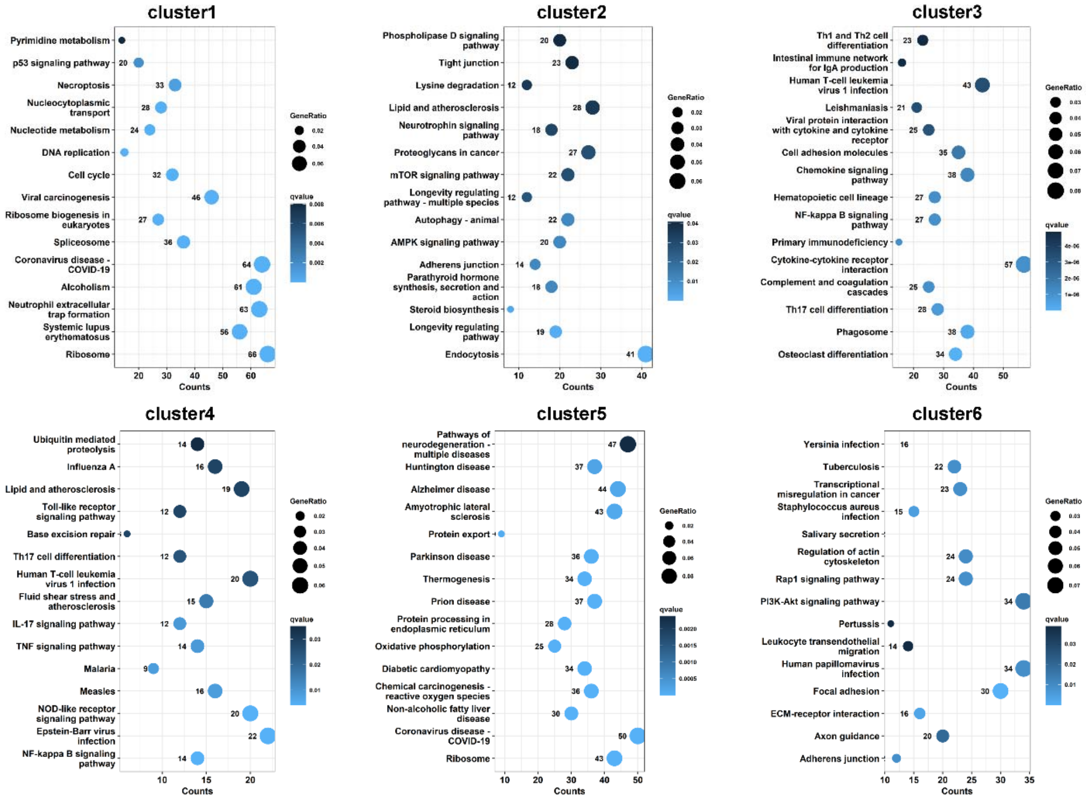


**Figure S15.** Kyoto Encyclopedia of Genes and Genomes (KEGG) pathway enrichment analysis of different cluster genes.

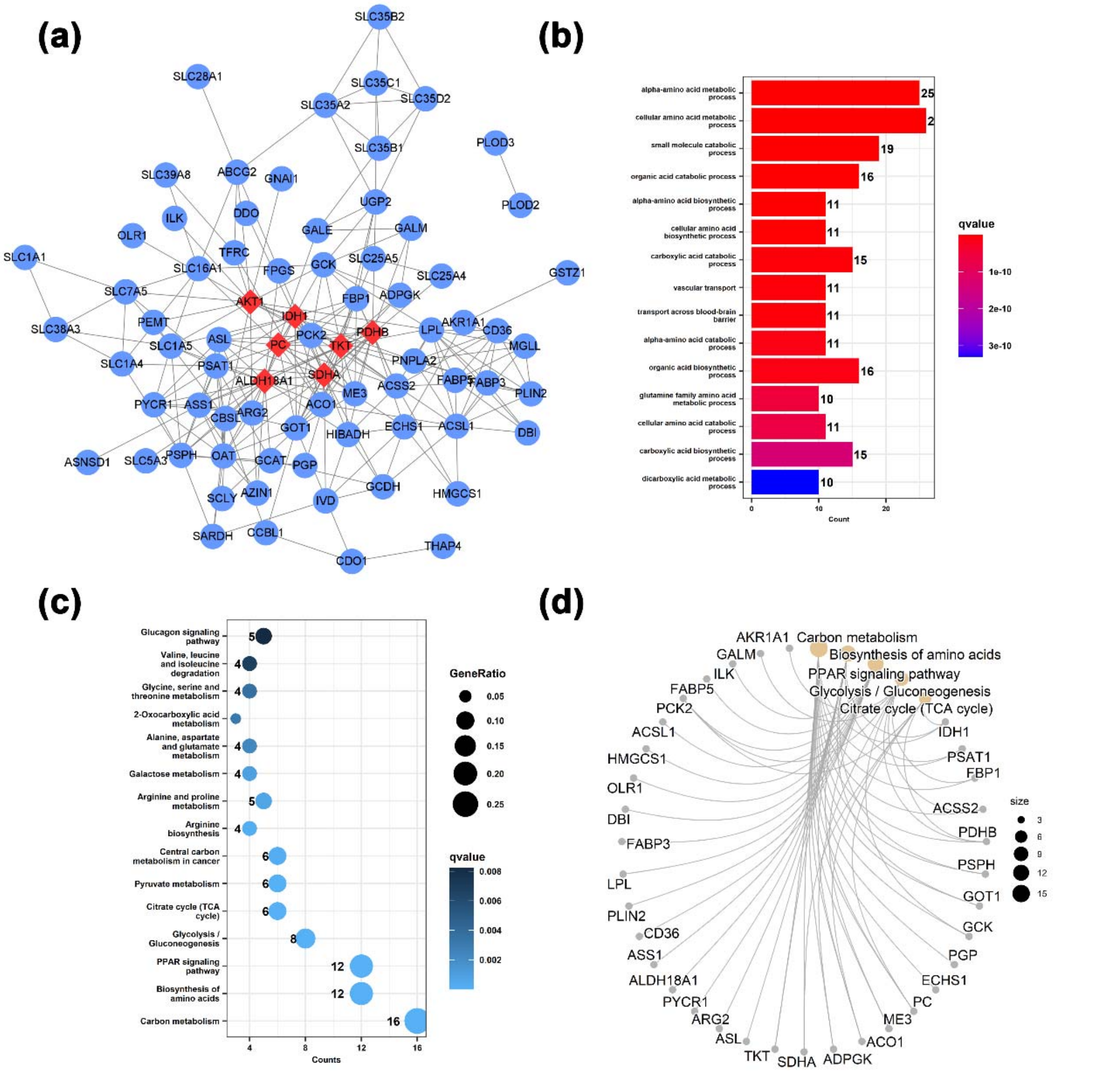


**Figure S16.** Gene function analysis related to substance metabolism. (**a**) Protein-protein interaction network analysis of metabolism-related genes. (**b**) Gene ontology enrichment analysis of metabolism-related genes. (**c**) Kyoto Encyclopedia of Genes and Genomes (KEGG) pathway enrichment analysis of metabolism-related genes. (**d**) Diagram of the relationship between genes and the KEGG signaling pathway.

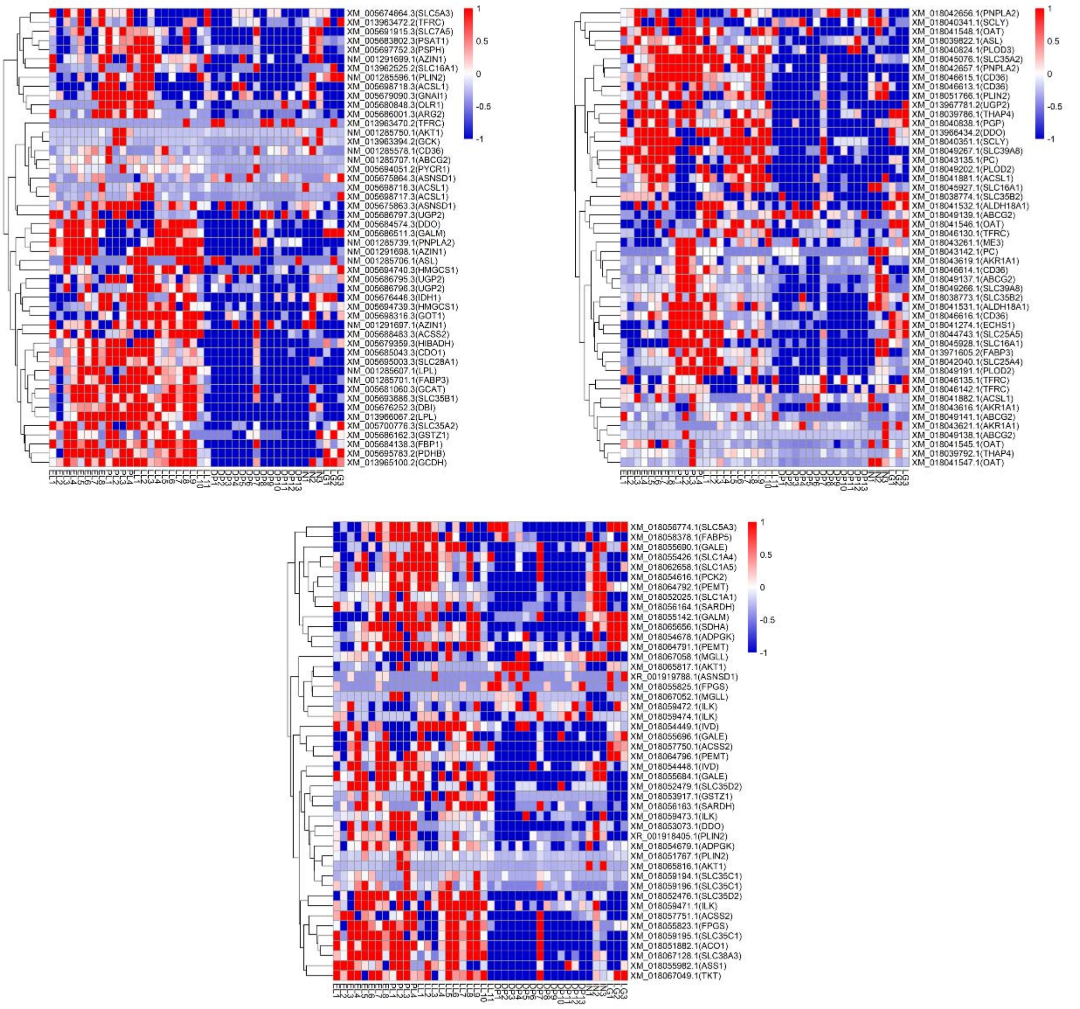

**Figure S17.** Expression heat map of metabolism-related genes at different developmental stages. EL: early lactation. PL: peak lactation. LL: late lactation. DP: dry period. IN: involution stage. LG: late gestation.

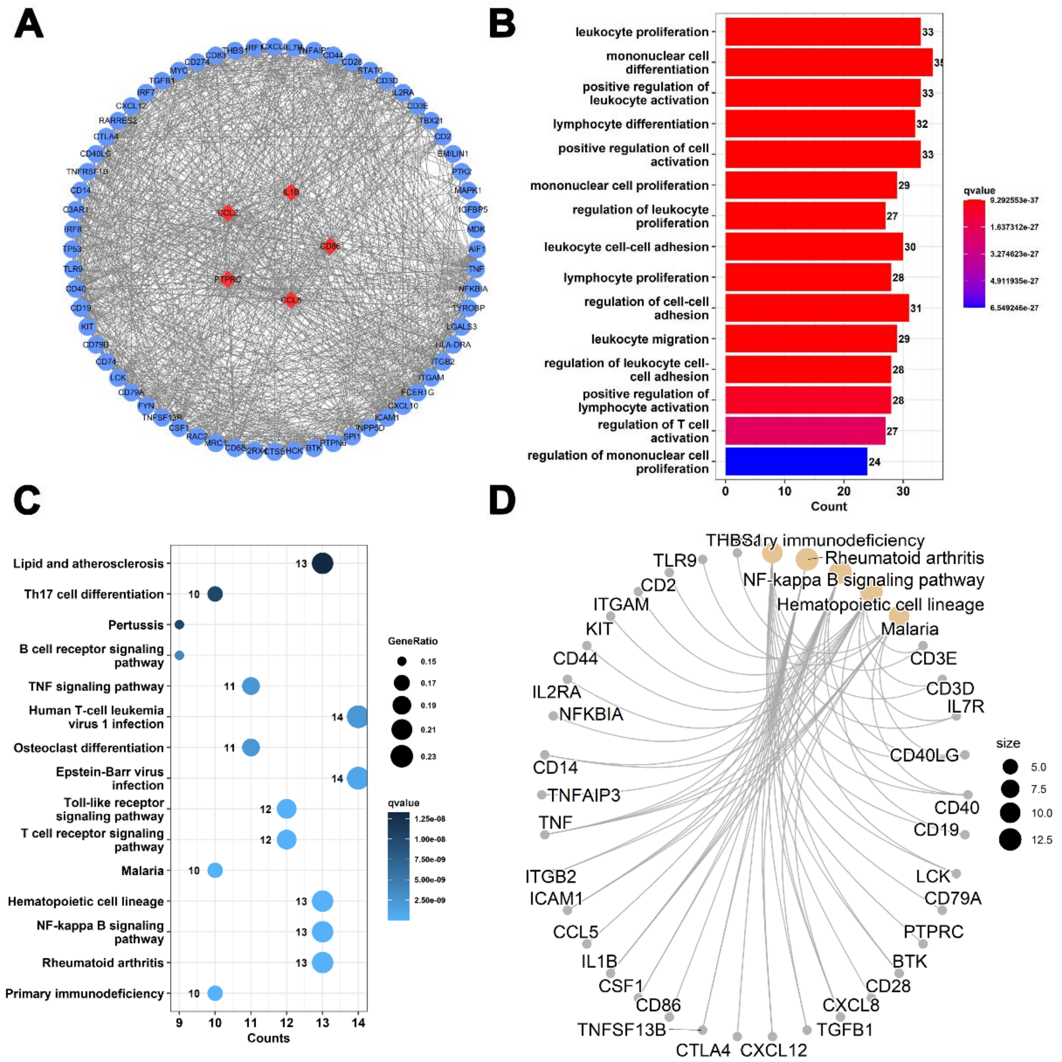


**Figure S18.** Gene function analysis related to immune-related genes. (**a**) Protein-protein interaction network analysis of immune-related genes. (**b**) Gene ontology enrichment analysis of immune-related genes. (**c**) Kyoto Encyclopedia of Genes and Genomes (KEGG) pathway enrichment analysis of immune-related genes. (**d**) Diagram of the relationship between genes and the KEGG signaling pathway.

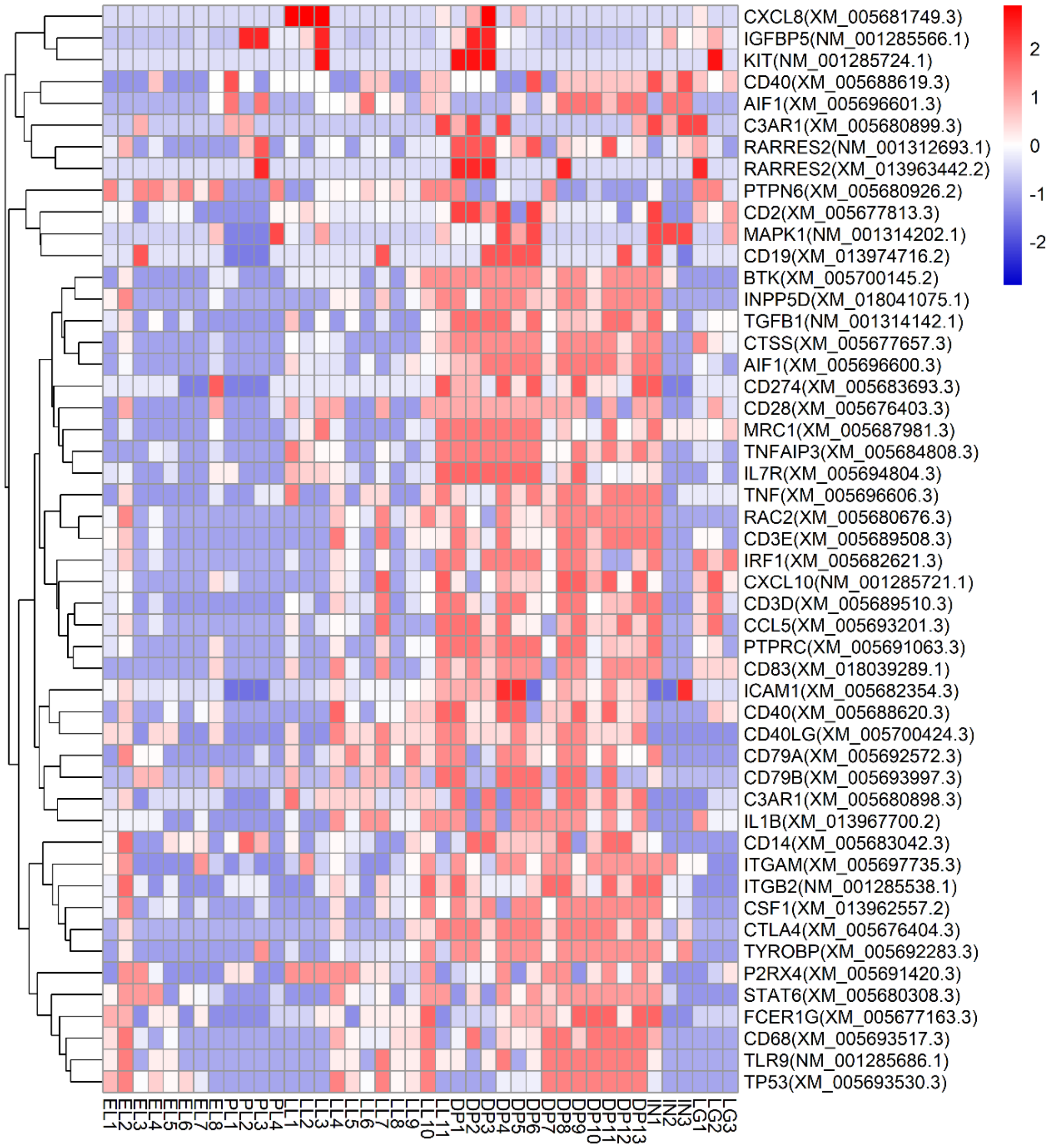


**Figure S19.** Expression heat map of immune-related genes at different developmental stages. EL: early lactation. PL: peak lactation. LL: late lactation. DP: dry period. IN: involution stage. LG: late gestation.

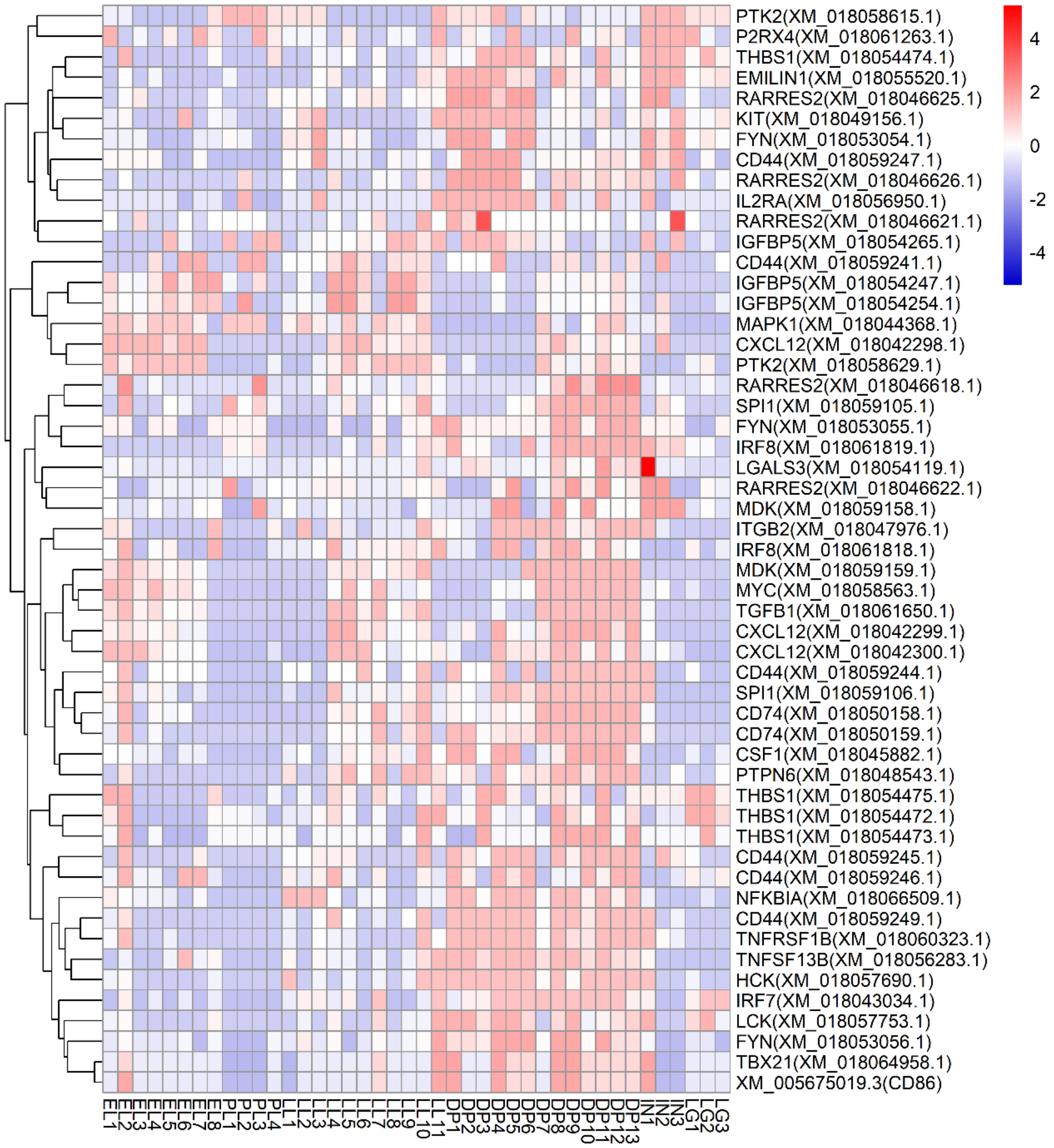


**Figure S20.** Expression heat map of immune-related genes at different developmental stages. EL: early lactation. PL: peak lactation. LL: late lactation. DP: dry period. IN: involution stage. LG: late gestation.

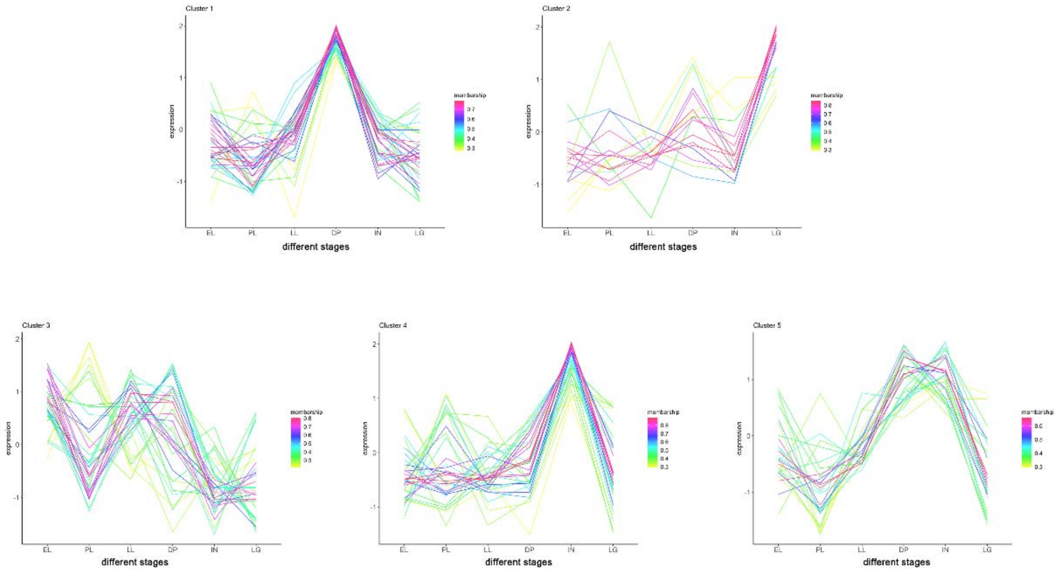


**Figure S21.** Expression cluster map of the members in the PI3K-Akt signaling pathway. A total of 146 transcripts (from 77 genes) were clustered into 5 clusters according to their expression levels.

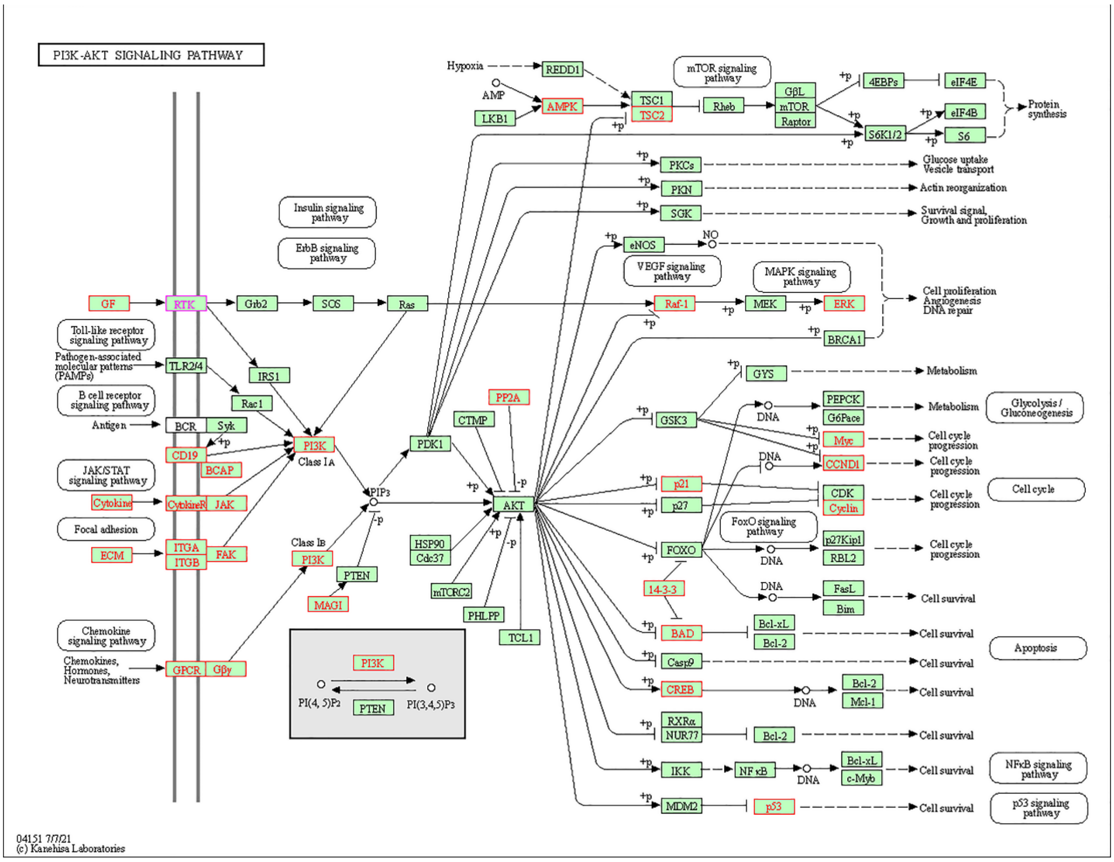


**Figure S22.** A map of PI3K-Akt signaling pathway.

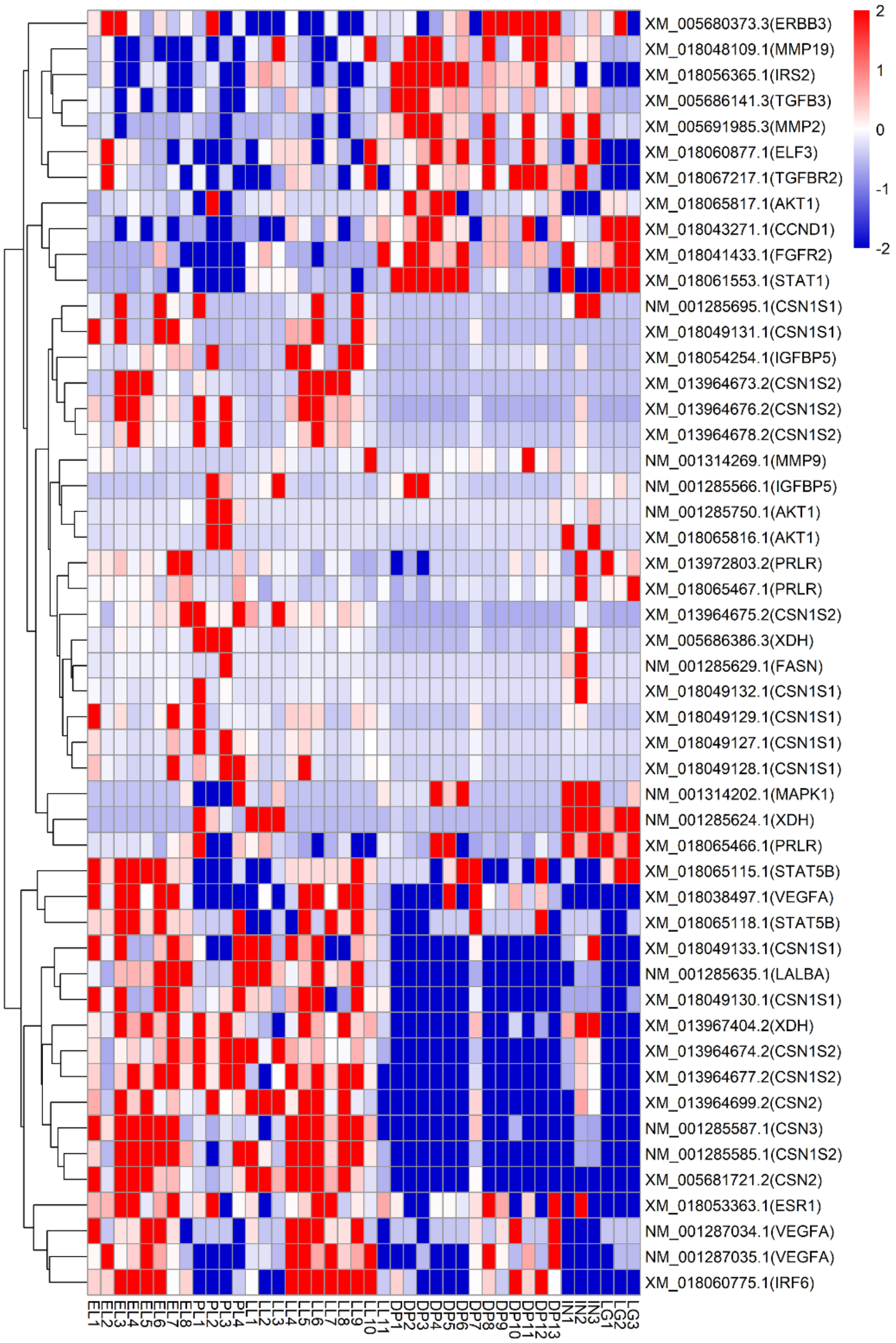


**Figure S23.** Heat map of expression of lactation and involution-related genes at different developmental stages. EL: early lactation. PL: peak lactation. LL: late lactation. DP: dry period. IN: involution stage. LG: late gestation.

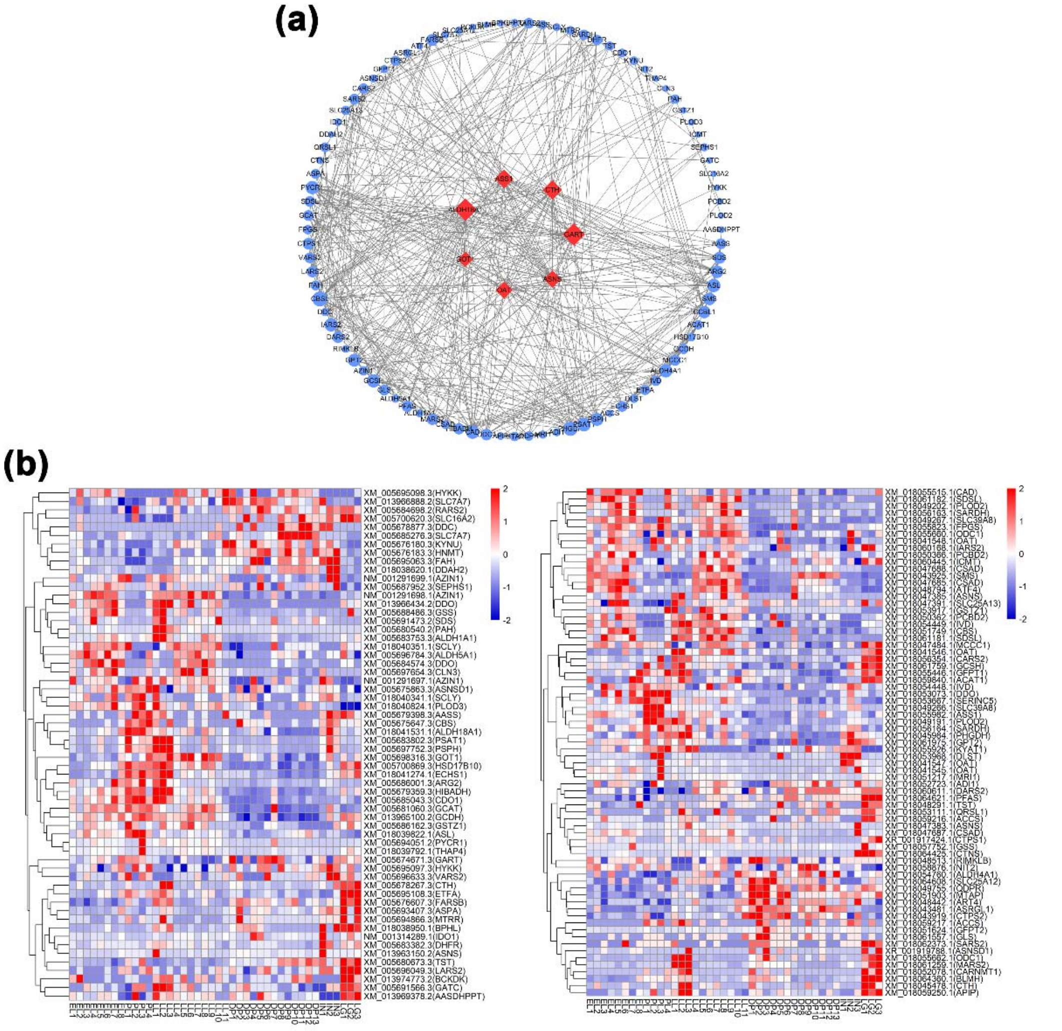


**Figure S24.** Analysis of genes related to protein synthesis and transport in mammary glands. (**a**) Protein-protein interaction network analysis of genes related to protein synthesis and transport in mammary glands. (**b**) Heat map of protein synthesis genes expression in different developmental stages.

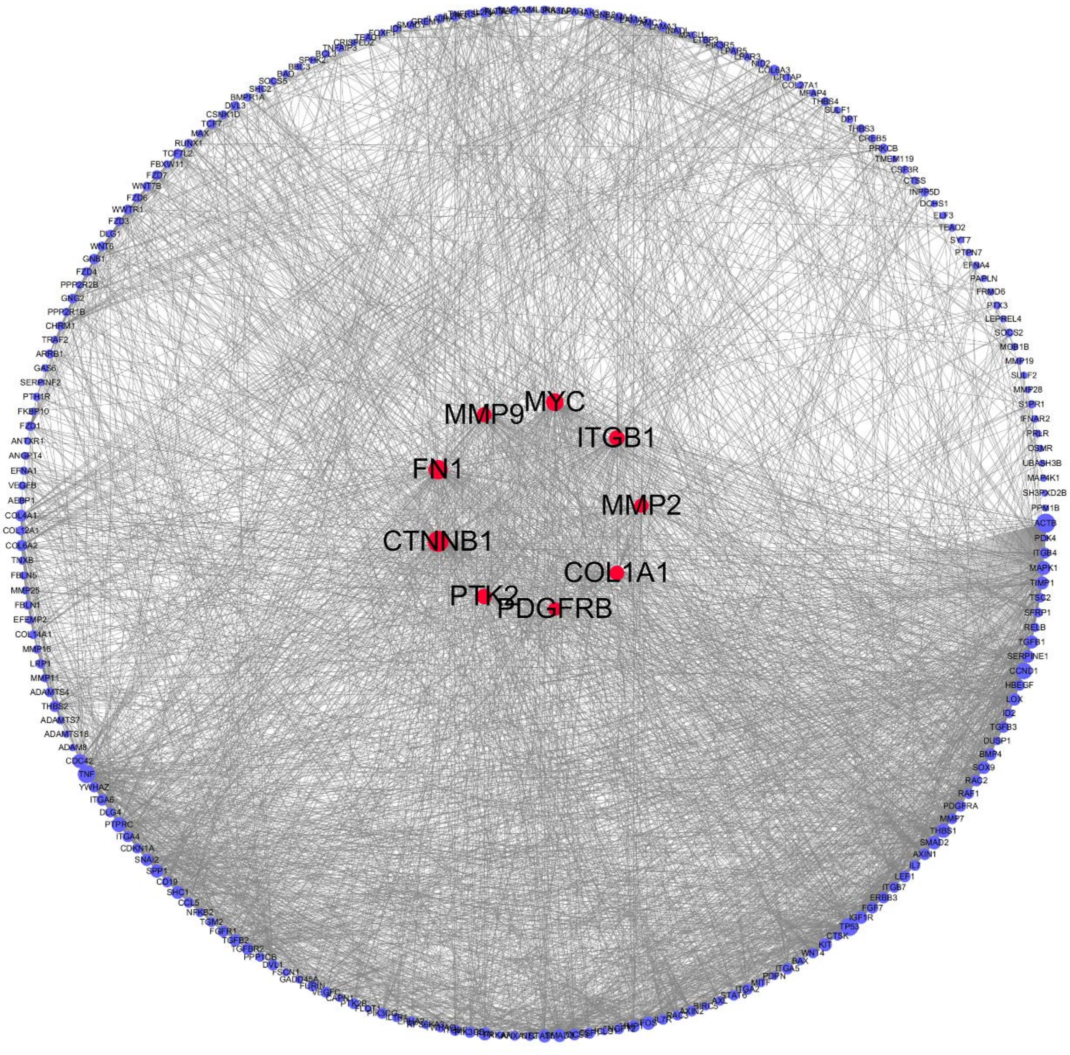


**Figure S25.** Protein-protein interaction network analysis of genes related to mammary gland development.

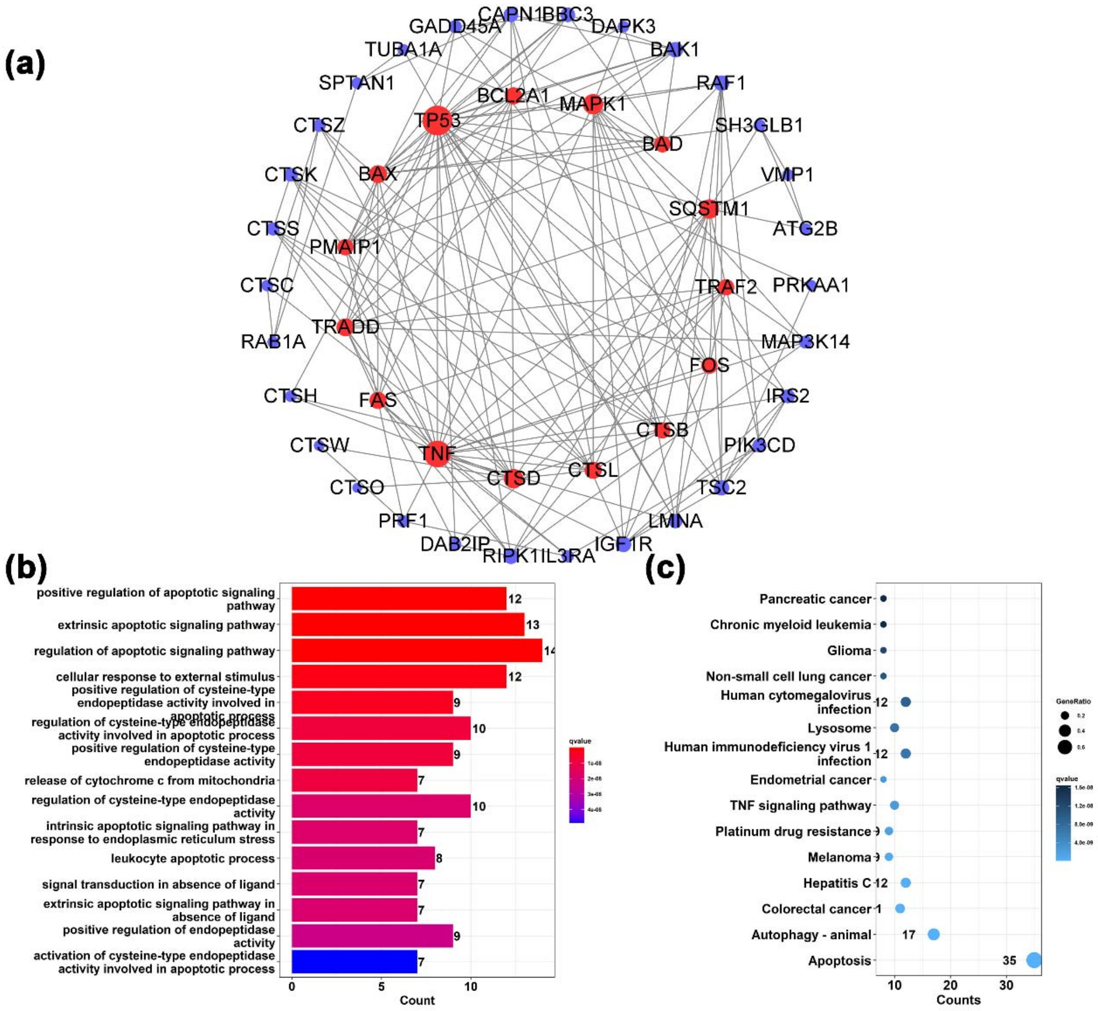


**Figure S26.** Analysis of genes related to apoptosis and autophagy in mammary glands. (**a**) Protein-protein interaction network analysis of genes related to apoptosis and autophagy. (**b**) Gene ontology enrichment analysis of genes related to apoptosis and autophagy. (**c**) Kyoto Encyclopedia of Genes and Genomes (KEGG) pathway enrichment analysis of genes related to apoptosis and autophagy.

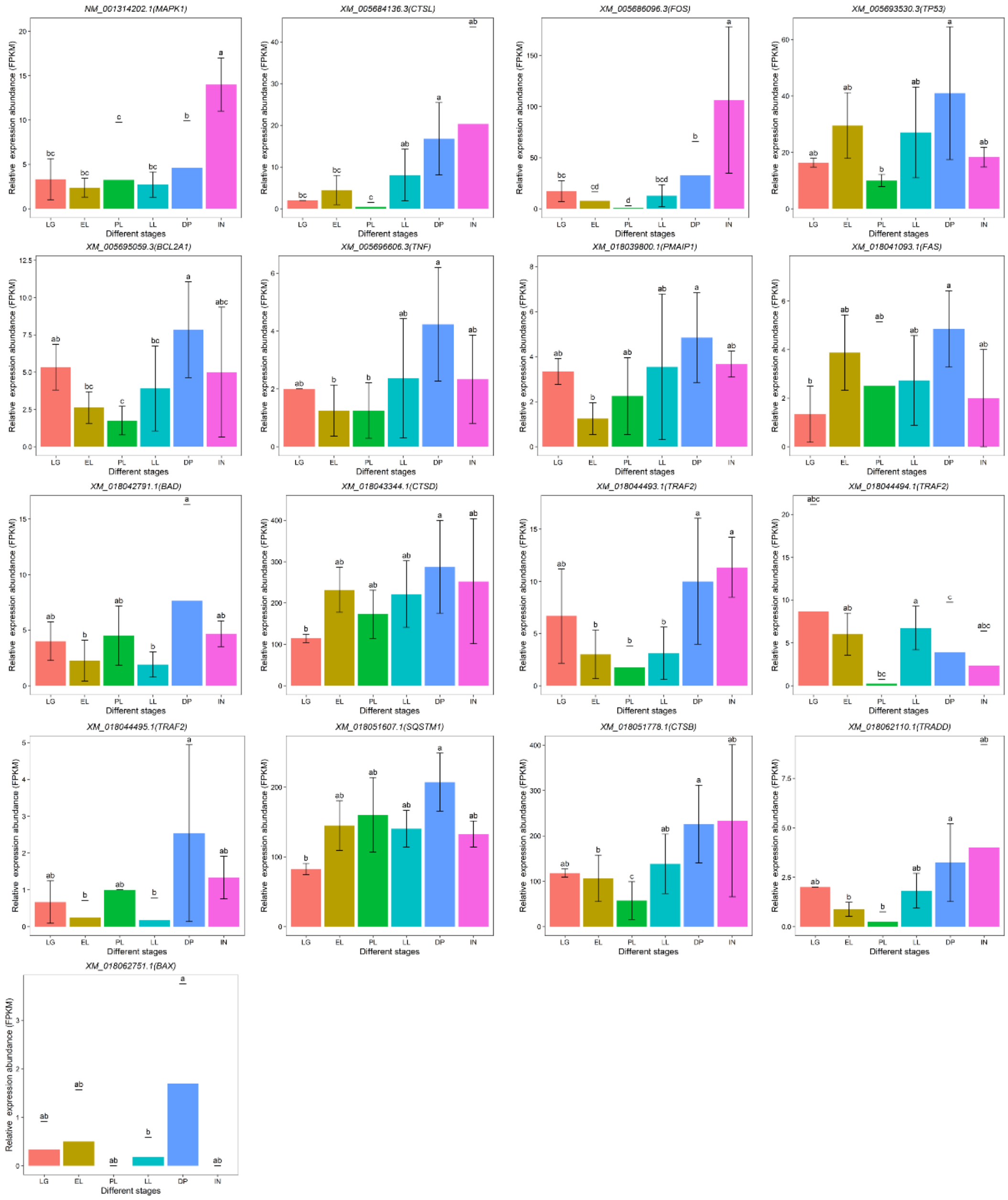


**Figure S27.** Bar graphs of expression patterns of apoptosis and autophagy-related hub genes at six mammary gland developmental stages.

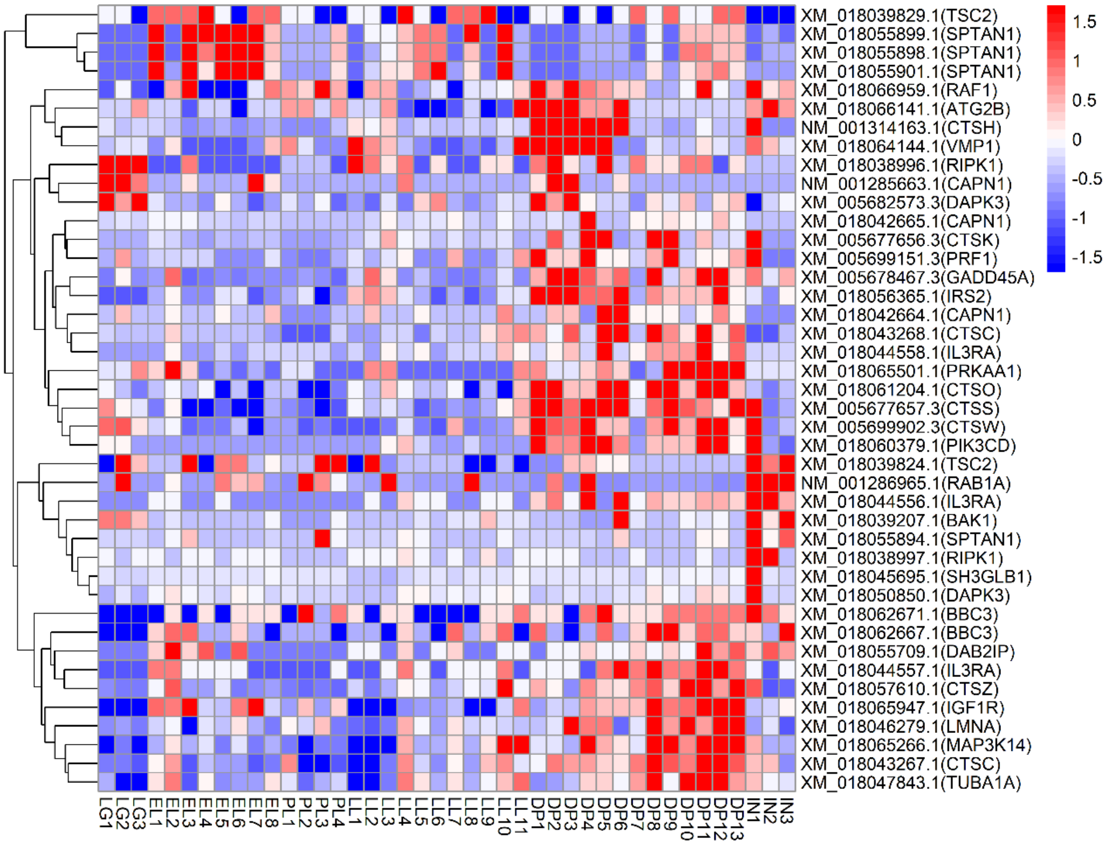


**Figure S28.** Expression heat map of apoptosis and autophagy-related genes at six mammary gland developmental stages.
