# Supplementary material for: Transcriptome Analysis of Goat Mammary Gland Tissue Reveals the Adaptive Strategies and Molecular Mechanisms of Lactation and Involution
Source: Int J Mol Sci. 2022 Nov 20;23(22):14424. doi: 10.3390/ijms232214424 (PMC9693614; doi:10.3390/ijms232214424)
Supplement: Supplementary file 1 [file ijms-23-14424-s001.zip › Supplementary Materials Tabels Titles.pdf]

Supplementary Materials Tables Title

Table S1. Primer sequences and amplification efficiency for real-time quantitative reverse-transcription PCR (RTqPCR).

Table S2. Source information and basic statistics of goat mammary glands samples for transcriptome analysis.

Table S3. Transcript expression levels of all mammary gland samples.

Table S4. Transcripts with expression in the top 20 at each developmental stage.

Table S5. Weighted gene co-expression network modules and nodes information.

Table S6. Gene ontology functional annotation and Kyoto Encyclopedia of Genes and Genomes (KEGG) pathway enrichment analysis of genes in each module

Table S7. Information on differentially expressed transcripts in mammary tissue at different developmental stages.

Table S8. Clustering results and Gene ontology and Kyoto Encyclopedia of Genes and Genomes (KEGG) pathway enrichment analysis of differentially expressed transcripts.

Table S9. Gene ontology and Kyoto Encyclopedia of Genes and Genomes (KEGG) pathway enrichment analysis of metabolism-related genes.

Table S10. Gene ontology and Kyoto Encyclopedia of Genes and Genomes (KEGG) pathway enrichment analysis of immune-related genes.

Table S11. Gene ontology and Kyoto Encyclopedia of Genes and Genomes (KEGG) pathway enrichment analysis of mammary gland development-related genes.

Table S12. Cluster analysis of genes in the PI3K-Akt signaling pathway.

Table S13. Gene ontology and Kyoto Encyclopedia of Genes and Genomes (KEGG) pathway enrichment analysis of genes related to apoptosis and autophagy.
